# LIN28A facilitates the transformation of human neural stem cells and promotes glioblastoma tumorigenesis through a pro-invasive genetic program

**DOI:** 10.18632/oncotarget.1131

**Published:** 2013-07-06

**Authors:** Xing-gang Mao, Marianne Hütt-Cabezas, Brent A. Orr, Melanie Weingart, Isabella Taylor, Anand K.D. Rajan, Yazmin Odia, Ulf Kahlert, Jarek Maciaczyk, Guido Nikkhah, Charles G. Eberhart, Eric H. Raabe

**Affiliations:** ^1^ Department of Pathology, Johns Hopkins University School of Medicine, Baltimore, MD; ^2^ Current address: Department of Neurosurgery, Xijing Hospital, Fourth Military Medical University, Xi'an, Shaanxi Province, China; ^3^ Department of Neurology, Johns Hopkins University School of Medicine, Baltimore, MD; ^4^ Current address: Neurologic Institute of New York, Columbia University, New York, NY; ^5^ Department of General Neurosurgery, Neurocenter, University Hospital Freiburg, Freiburg, Germany; ^6^ Current address: Department of Stereotactic and Functional Neurosurgery, University Medical Center Duesseldorf, Germany; ^7^ Department of Stereotactic and Functional Neurosurgery, Neurocenter, University Hospital Freiburg, Freiburg, Germany; ^8^ Current address: Clinic of Neurosurgery, University Medical Center Erlangen, Germany; ^9^ Division of Pediatric Oncology, Johns Hopkins University School of Medicine, Baltimore, MD

**Keywords:** let-7, stem cell, microRNA, HMGA2, SNAI1

## Abstract

The cellular reprogramming factor *LIN28A* promotes tumorigenicity in cancers arising outside the central nervous system, but its role in brain tumors is unknown. We detected LIN28A protein in a subset of human gliomas observed higher expression in glioblastoma (GBM) than in lower grade tumors. Knockdown of LIN28A using lentiviral shRNA in GBM cell lines inhibited their invasion, growth and clonogenicity. Expression of LIN28A in GBM cell lines increased the number and size of orthotopic xenograft tumors. LIN28A expression also enhanced the invasiveness of GBM cells *in vitro* and *in vivo*. Increasing LIN28A was associated with down-regulation of tumor suppressing microRNAs *let-7b* and *let-7g* and up-regulation of the chromatin modifying protein HMGA2. The increase in tumor cell aggressiveness *in vivo* and *in vitro* was accompanied by an upregulation of pro-invasive gene expression, including *SNAI1*. To further investigate the oncogenic potential of *LIN28A*, we infected hNSC with lentiviruses encoding *LIN28A* together with dominant negative *R248W-TP53*, constitutively active *KRAS* and *hTERT*. Resulting subclones proliferated at an increased rate and formed invasive GBM-like tumors in orthotopic xenografts in immunodeficient mice. Similar to *LIN28A*-transduced GBM neurosphere lines, hNSC-derived tumor cells showed increased expression of HMGA2. Taken together, these data suggest a role for *LIN28A* in high grade gliomas and illustrate an *HMGA2*-associated, pro-invasive program that can be activated in GBM by *LIN28A*-mediated suppression of *let-7* microRNAs.

## INTRODUCTION

*LIN28A* is a microRNA-regulating protein linked to a variety of cancers, including breast, prostate, and gastic carcinoma [1-4]. *LIN28A* and its homolog *LIN28B* are expressed in human tumors that are poorly differentiated and carry the worst prognosis [5, 6]. The primary known targets of LIN28A and the related protein LIN28B are the *let-7* family of tumor suppressing microRNAs.

These microRNAs suppress the translation of critical mediators of stemness, proliferation, and invasion such as KRAS, c-MYC, and HMGA2 [7-10]. Over-activation of LIN28A or LIN28B is one mechanism by which cancer cells can eliminate *let-7* microRNAs and allow for increased expression of pro-oncogenic signals [5]. *HMGA2* is a stem cell factor that promotes the invasiveness of cancer cells [11, 12]. *HMGA2* directly binds to the promoter and regulates the pro-invasion transcription factor *SNAI1* (*SNAIL*) as well as other pro-invasion targets [13, 14].

LIN28A expression is high during the early stages of neural tube development and decreases over time [15]. Recent papers have described increased expression of LIN28A in primitive neuro-ectodermal tumors and atypical teratoid rhaboid tumors, which are aggressive pediatric brain tumors [16, 17]. However, the functional role of LIN28A in brain neoplasms has not been explored. The established role of LIN28A in neural development and the importance of NF1/RAS/MEK/ERK and MYC signaling in GBM led us to hypothesize that *LIN28A* would promote GBM tumorigenesis. In this study we use human primary tumor samples, GBM-derived human cell lines and human neural stem cells to investigate the role of *LIN28A* in malignant gliomas *in vitro* and *in vivo*.

## RESULTS

### LIN28A and LIN28B are expressed in human glioma samples

**Figure 1:**
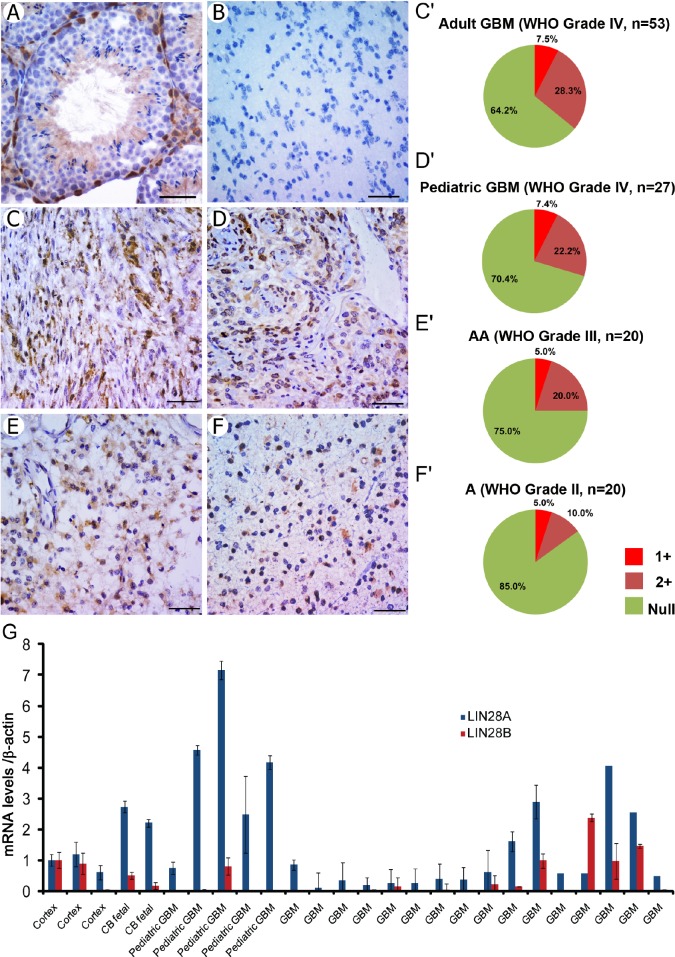
LIN28A protein is expressed in human primary glioma samples, with highest expression in GBM and anaplastic astrocytoma LIN28A expression in glioma tissue microarrays was determined by immunohistochemistry. A. Positive control for LIN28A expression in mouse testis (400X magnification). The basal spermatogonia are positive, but more mature cells and supporting cells in the testis are negative. B. LIN28A protein is not detected in normal human adult brain tissue (400X). C-F. LIN28A protein is expressed at strong or moderate levels in approximately 40 percent of adult GBM (400X, C and pie chart C'), 25 percent of pediatric GBM (400X, D, pie chart D'), 25 percent of WHO grade III anaplastic astrocytoma (400X, E, pie chart E') and 15 percent of WHO grade II astrocytoma (400X, F;F'). Bar= 50 μm. G. *LIN28A* and *LIN28B* mRNA levels in primary GBM samples detected by qPCR, showing that a subset of adult and pediatric GBM express *LIN28A* or *LIN28B*. Normal adult human cortex and fetal human cerebellum (CB) samples are included at left for comparison.

To investigate the potential role of *LIN28A* in gliomas, we examined LIN28A protein by immunohistochemistry in tissue microarrays (TMA) containing human glioma and GBM samples, including 20 astrocytomas (WHO grade II), 20 anaplastic astrocytomas (WHO grade III, AA), 35 pediatric glioblastoma (WHO grade IV, pGBM) and 64 adult glioblastoma (WHO grade IV, aGBM). We confirmed the specificity of the antibody by showing that LIN28A was present only in mouse testis spermatagonia and not more mature germ cells, as previously reported [30] (Figure 1A). We did not detect LIN28A expression in normal human brain (Figure 1B). All grades of glioma expressed LIN28A with varying intensity of immunostaining (Figure 1C-F). Positive reactivity was observed in 15% of grade II astrocytoma, 25% of grade III anaplastic astroctyoma, 23% of pediatric GBM and 44% of adult GBM. Most of the LIN28A-positive tissue samples showed moderate intensity staining, but approximately 5% of cases showed strong reactivity. Analysis of primary pediatric and adult GBM by qPCR showed that a subset (42%) expressed mRNA levels of *LIN28A* or the related gene *LIN28B* in excess of normal brain (Figure 1G). Only one tumor expressed both *LIN28A* and *LIN28B* at levels greater than normal brain.

### *LIN28A* expression positively correlates in GBM with the stem cell and pro-invasion factors *HMGA2* and *SNAI1* and the stem cell factor *OCT4*

**Figure 2:**
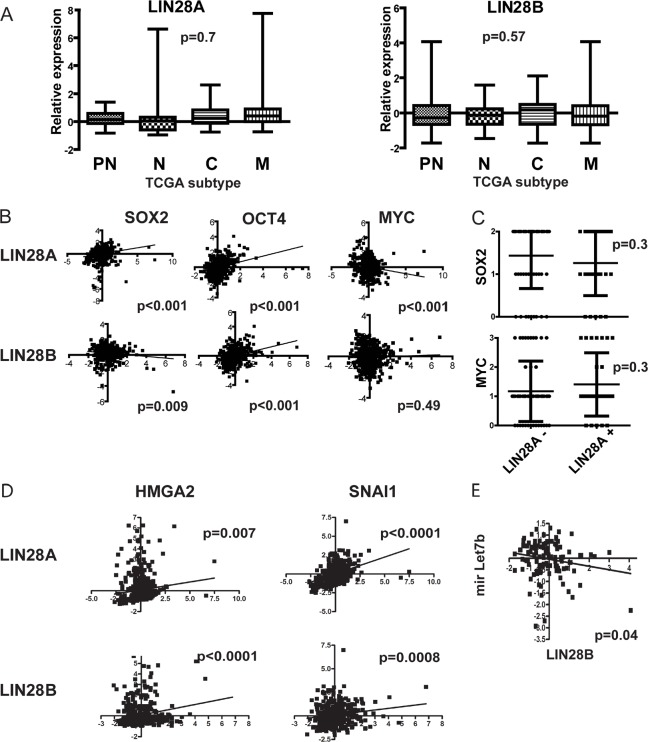
Interrogation of The Cancer Genome Atlas (TCGA) dataset of more than 500 GBM tumors shows that *LIN28A* and *LIN28B* are associated with a pro-invasive program and also with some cancer stem cell genes A. Box-and-whisker plots of TCGA subsets shows that *LIN28A* and *LIN28B* expression is highly variable across GBM and is not associated with any particular GBM subtype. P values are as determined by ANOVA with multiple comparisons correction. PN= proneural, N= neural, C= classical, M = mesenchymal. B. At the mRNA level, within the TCGA dataset, there is a positive correlation between *LIN28A* expression (x-axis) and the expression of the neural stem cell markers *SOX2* and *OCT4* (y-axis). Although there is a similar positive correlation between *LIN28B* and *OCT4*, there is a negative correlation between *LIN28B* and *SOX2*. There is a negative correlation between *LIN28A* expression and *MYC* and no significant correlation between *LIN28B* and *MYC*. The correlation of expression values was determined using Pearson's correlation coefficient. C. Vertical dot plot analysis of a tissue microarray of 80 pediatric and adult GBM for LIN28A, MYC and SOX2 protein expression by immunohistochemistry shows no significant clustering of SOX2 or MYC expression in tumors expressing LIN28A. D. Analysis of TCGA data shows correlation of *LIN28A* and *LIN28B*(x-axis) with the stem cell and pro-invasion factors *HMGA2* and *SNAI1*(y-axis). E. There is an inverse correlation between LIN28B and *let7b* expression.

To further quantify *LIN28A* and *LIN28B* expression in high-grade gliomas, we expanded our evaluation of GBM to include The Cancer Genome Atlas (TCGA) dataset containing more than 500 GBM samples. In TCGA data, similar to our mRNA analysis of local samples, a subset of GBM express *LIN28A* or *LIN28B*. However, neither *LIN28A* nor *LIN28B* expression segregated within any of the TCGA GBM subgroupings (Figure 2A). Although both *LIN28A* and *LIN28B* were correlated at the mRNA level with the stem cell factor *OCT4*, only *LIN28A* positively correlated with expression of the neural stem cell factor *SOX2* (Figure 2B). When we stained our 80 tumor tissue microarray for SOX2 and LIN28A, we determined that there was no correlation between positivity for LIN28A and SOX2 at the protein level (p=0.58, Pearson correlation) (Figure 2C). We also did not observe any positive correlation between *LIN28A* and *LIN28B* and *MYC* at the mRNA level in TCGA dataset or by IHC between LIN28A and MYC on our 80 GBM tumor tissue microarray (Figure 2B, 2C).

In contrast, we did observe statistically significant positive correlations in TCGA dataset between *LIN28A* or *LIN28B* and the stem cell and pro-invasion factors *HMGA2* and *SNAI1* (Figure 2D). There was a negative correlation between *LIN28B* and the HMGA2 suppressing microRNA *let-7b* (Figure 2E). In total, 18 percent of GBM in TCGA dataset show increased expression of *LIN28A*, *LIN28B* or the LIN28A/LIN28B regulated gene *HMGA2*.

### Knockdown of *LIN28A* in GBM cell lines decreases invasion, proliferation and clonogenicity

**Figure 3:**
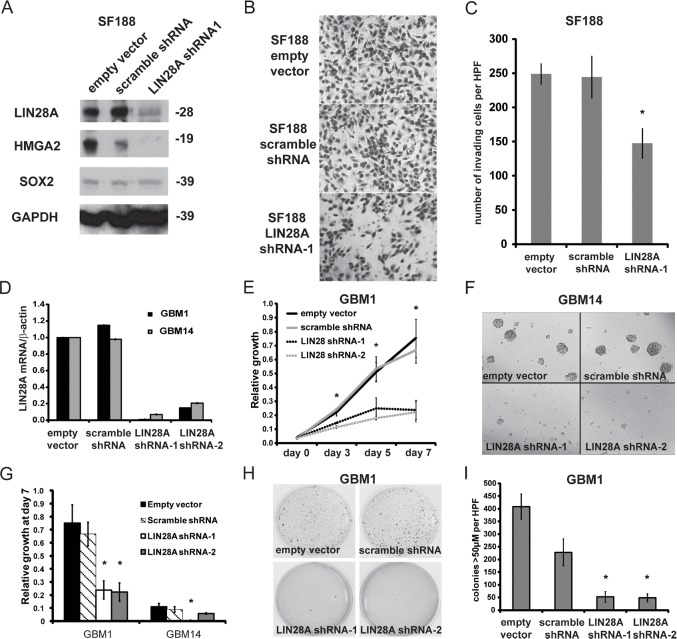
Knockdown of *LIN28A* in GBM neurosphere cell lines inhibits their invasion, growth and colony formation A. Short-term infection of the SF188 pediatric GBM cell line with lentiviral LIN28A-shRNA leads to efficient knockdown of LIN28A protein expression compared to control pLKO (empty vector) and scramble-shRNA constructs. The *let-7* regulated, pro-invasion factor HMGA2 is also downregulated, but the neural stem cell factor SOX2 is not affected by suppression of LIN28A knockdown. B. Photomicrographs of Matrigel Boyden chamber invasion assay showing that lentiviral LIN28A-shRNA suppresses the invasion of SF188 cells compared to control pLKO or scramble-shRNA constructs. Power = 200X. C. Quantification of a representative experiment. Student's t-test * = p<0.005, pLKO or scramble shRNA versus LIN28A-shRNA. Results were repeated twice more with similar findings. D. LIN28A-shRNA leads to decreased *LIN28A* mRNA levels in HSR-GBM1 and JHH-GBM14 compared to empty vector or scramble shRNA. E. MTS assay showing that LIN28A knockdown by shRNA infection in HSR-GBM1 leads to decreased growth. Asterisks indicate p< 0.05 by one way ANOVA compared to control pLKO and scramble. F. Representative images of neurospheres from the MTS assay at day 7 showing that *LIN28A* knockdown in JHH-GBM14 leads to decreased neurosphere size. G. Quantification of MTS assay at day 7 showing that LIN28A knockdown in both HSR-GBM1 and JHH-GBM14 decreases the growth of these cell lines. ANOVA, *p<0.05 compared to empty vector and scramble. H. Representative wells of soft agarose assay comparing HSR-GBM1 empty vector and scramble shRNA to two *LIN28A*-specific shRNAs, showing decreased colony formation in *LIN28A*-shRNA transduced cells. I. Quantification of colony numbers shown in H, demonstrating that LIN28A knockdown in HSR-GBM1 significantly inhibits colony formation in soft agarose. ANOVA, *p<0.05 compared to HSR-GBM1-empty vector and GBM1-scramble.

To investigate the importance of *LIN28A* in GBM, we performed *LIN28A* knockdown with LIN28A-shRNA lentivirus in lines derived from pediatric (SF188) and adult GBM (HSR-GBM1 and JHH-GBM14). LIN28A-shRNA effectively decreased expression of LIN28A as measured by western blot in SF188 (Figure 3A). LIN28A knockdown in SF188 cells led to a more than 40% decrease in invasion as measured by transwell invasion assay (Figure 3B, C).

In adult GBM, LIN28A-shRNA decreased *LIN28A* mRNA by approximately 85% in HSR-GBM1, and 93% in JHH-GBM14 compared to empty vector and non-targeting scramble shRNA as determined by qPCR (Figure 3D). The growth of HSR-GBM1 and JHH-GBM14 was significantly inhibited after *LIN28A* knockdown as demonstrated by MTS assays (Figure 3E-G). Soft agarose assay was performed to examine the clonogenic potential of HSR-GBM1 after *LIN28A* knockdown. HSR-GBM1 LIN28A-shRNA transduced cells formed significantly fewer colonies in soft agarose compared to control GBM1 pLKO empty-vector and GBM1-scramble transduced cells. (Figure 3H, I; p<0.05 by ANOVA).

### Expression of *LIN28A* in GBM neurosphere cell lines promotes tumor formation *in vivo*

**Figure 4:**
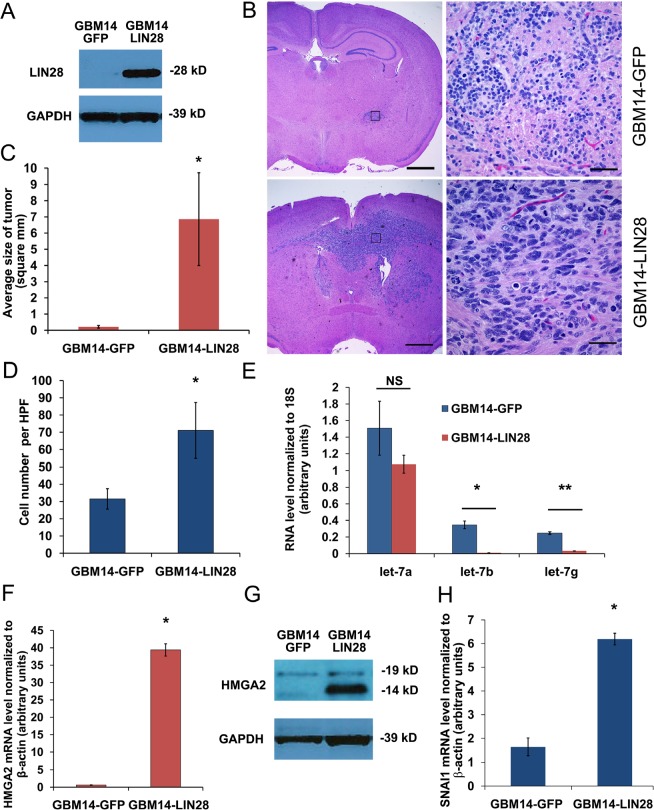
Introduction of *LIN28A* into the JHH-GBM14 stem cell line activates a pro-invasion program and promotes tumor formation *in vivo* A. Western blot showing increased LIN28A expression in GBM14 infected with *LIN28A* lentivirus. B. Low power (40X) magnification showing increased tumor size of a representative JHH-GBM14-LIN28A xenograft (bottom panels) compared to control JHH-GBM14-GFP xenograft (top panels). C. Quantification of tumor size based on largest diameter in two dimensions. Asterisk p=0.056 Student's *t-*test. D. Quantification of Matrigel Boyden chamber invasion assay showing increased invasiveness of JHH-GBM14-LIN28A compared to JHH-GBM14-GFP control. Asterisk = p<0.05 Student's *t-*test. E. qPCR showing that *let-7b* and *let-7g*, but not *let-7a*, are downregulated in JHH-GBM14-LIN28A compared to JHH-GBM14-GFP control. F. qPCR showing that HMGA2 mRNA is increased in JHH-GBM14-LIN28A compared to JHH-GBM14-GFP control. G. Western blot showing that HMGA2 protein is increased in JHH-GBM14-LIN28A compared to JHH-GBM14-GFP control. H. qPCR showing increased mRNA expression of *SNAI1* in JHH-GBM14-LIN28A compared to JHH-GBM14-GFP control.

Next we tested if expression of *LIN28A* would promote xenograft tumor formation in GBM neurosphere cell lines. We chose two human GBM cell lines, 040622 [19] and JHH-GBM14 [20], which are poorly tumorigenic after orthotopic injection into immunodeficient mice, and infected them with *LIN28A* lentivirus or GFP control. Expression of LIN28A was confirmed by western blotting (Figure 4A, Supplemental Figure 1A).

These cells were injected into the brains of nude mice to test their tumorigenicity. Within one month of injection of 040622-LIN28A, large tumors formed in all mice (n= 5), while 040622-GFP mice (n=5) did not show signs of tumor formation at that time (p= 0.0079, Fisher's exact test) (Supplemental figure 1B). After 3 months, 040622-GFP injected mice remained asymptomatic. These mice were sacrificed and 100 percent had small, non-invasive tumors.

For JHH-GBM14, both GFP and LIN28A injected groups were sacrificed four months after intracranial injection to examine tumor formation. We identified large, invasive brain tumors in 6/8 (75%) of JHH- GBM-14 LIN28A injected animals, while control JHH-GBM-14-GFP injected animals had small tumors in 2/7 (20%) of injected animals (Figure 4B). The JHH-GBM-14 LIN28A tumors were substantially bigger than control tumors (Figure 4B, C). In addition to forming brain tumors larger than JHH-GBM14-GFP controls, JHH-GBM14-LIN28A tumors significantly invaded into normal tissues including the corpus callosum and the contralateral brain hemisphere (Figure 4B). These tumors expressed LIN28A, were SOX2, Nestin and GFAP positive and Synaptophysin negative, indicating that their immunoprofile remained consistent with GBM phenotype (Supplemental Figure 1C). In an *in vitro* correlate of the *in vivo* findings, more JHH-GBM14-LIN28A cells migrated and invaded through Matrigel in a transwell chamber assay than control JHH-GBM14-GFP cells (Figure 4D).

**Figure 5:**
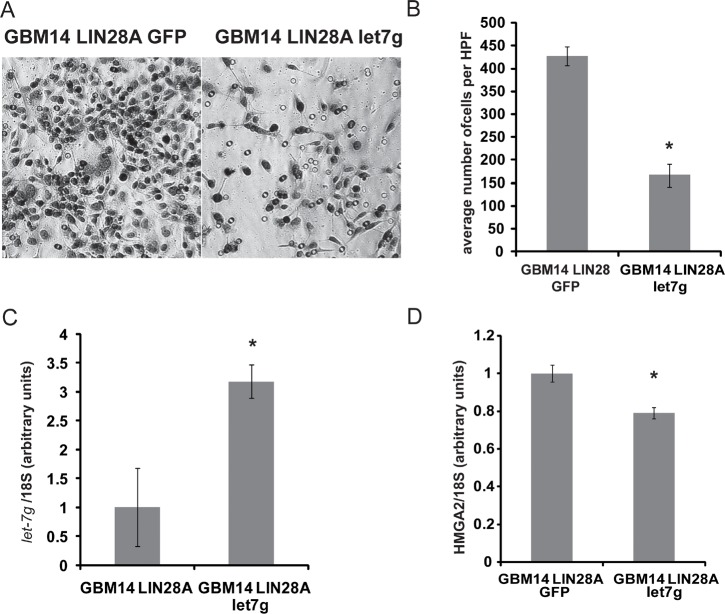
Expression of mature *let-7g* can reverse the pro-invasive phenotype of LIN28A expressing GBM14 cells GBM14 LIN28A expressing cells were nucleofected with either GFP or *let7g* expression plasmids and 48 hours later were placed into a Boyden transwell migration chamber. A. High power view (200X) of migrated cells 48 hours after plating in the upper chamber shows decreased migration in GBM14/LIN28A/*let-7g* expressing cells (right) compared to GBM14/LIN28A/GFP expressing cells left. B. Graph showing quantification of migration. Asterisk = p<0.0005 Student's *t-test* GBM14/LIN28A/*let-7g* versus GBM14/LIN28A/GFP. C. qPCR showing increased expression of *let-7g* in *let-7g­-*nucleofected cells compared to GFP-nucleofected cells. Asterisk = p<0.01, Student's *t-*test GBM14/LIN28A/*let-7g* versus GBM14/LIN28A/GFP. D. qPCR showing decreased expression of *HMGA2* in *let-7g­-*nucleofected cells compared to GFP-nucleofected cells. Asterisk = p<0.01, Student's *t-*test GBM14/LIN28A/*let-7g* versus GBM14/LIN28A/GFP.

To investigate the potential mechanisms of LIN28A-induced tumorigenicity, we detected the expression levels of *let-7* microRNA and known *let-7* targets in JHH-GBM14-LIN28A and JHH-GBM14-GFP. Levels of *let-7b* and *let-7g* decreased in LIN28A-transduced cells, compared to GFP controls (Figure 4E), while *let-7a* levels did not change significantly. HMGA2, a known *let-7* target, was upregulated in GBM14-LIN28A (Figure 4F, G) compared to GBM14-GFP. We examined the expression of genes involved in promoting invasion, and we identified increased *SNAI1*, a zinc finger transcription factor and key regulator of invasion, in JHH-GBM14-LIN28A cells compared to JHH- GBM14-GFP (Figure 4H). Transduction of GBM14-LIN28A cells with mature *let-7g* led to reversal of the invasive phenotype and a corresponding downregulation of HMGA2 (Figure 5A-D).

### *LIN28A* facilitates *KRAS*-mediated transformation of human neural stem cells

**Figure 6:**
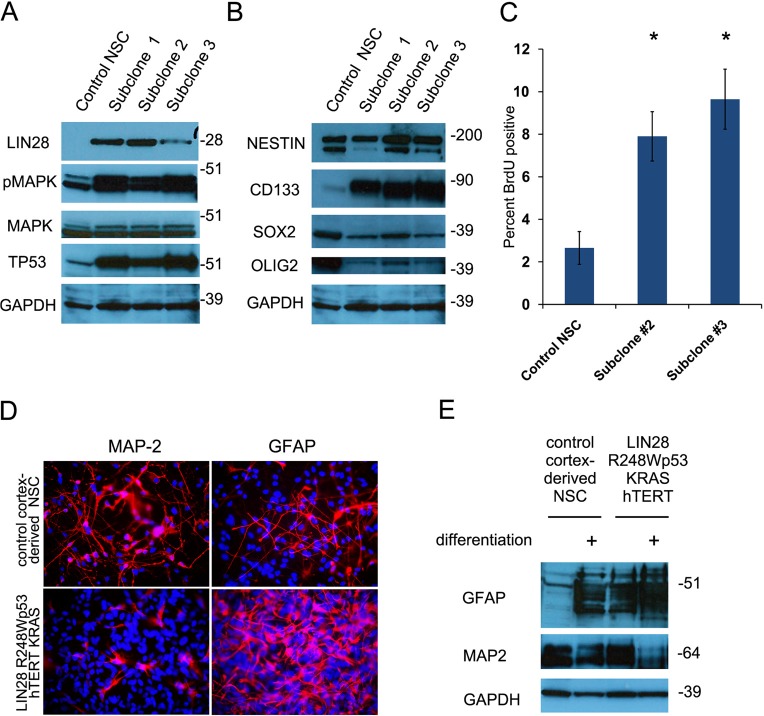
Transduction of human neural stem cells with *LIN28A* and other oncogenic elements promotes proliferation and disrupts normal differentiation A. Western blot showing increased expression of LIN28A, TP53 and increased phospho-MAP kinase expression in human neural stem cells transduced with lentiviruses containing *R248WTP53*, CA-*KRAS*, *hTERT* and *LIN28A*. B. Western blot showing continued expression of neural stem cell markers such as CD133, SOX2, OLIG2, and Nestin by hNSC-DNp53/hTERT/KRAS/LIN28A. C. hNSC-DNp53/hTERT/KRAS/LIN28A show increased proliferation compared to normal hNSC as determined by BrdU incorporation assay (7.9 and 9.6% BrdU positive cells in two separate subclones) compared to normal hNSCs (2.7±1.6% BrdU positive cells, *p*<0.05 Student's *t-*test). D. Immunofluorescence photomicrograph showing that after differentiation in 2% serum medium for 14 days, hNSC DNp53/hTERT/KRAS/LIN28A cells exhibit increased expression of the glial marker GFAP and decreased expression of the neuronal marker MAP2, compared to control cortical hNSC. E. Western blot showing that hNSC-DNp53/hTERT/KRAS/LIN28A cells express more glial marker GFAP at baseline and after differenentiation, compared to control cortical neural stem cells. There is a corresponding decrease in MAP2 expression after differentiation, consistent with the changes observed in the immunofluorescence images in D.

We further examined if LIN28A in conjunction with other oncogenes could transform normal cells growing in culture. We infected cortex-derived human neural stem cells (hNSC) with lentiviruses encoding LIN28A together with dominant negative R248WTP53, constitutively active KRAS (CA-KRAS) and hTERT (hereafter referred to as hNSC- LIN28A/DNp53/hTERT/KRAS), all of which are known to be oncogenic elements in GBM [31-33]. As controls, hNSC were also infected with DNp53/hTERT/KRAS, LIN28A/DNp53/hTERT, DNp53/hTERT, or with GFP. Compared to control normal hNSCs, LIN28A/DNp53/hTERT/KRAS transduced hNSCs had increased LIN28A, TP53, and phospho-MAPK expression by western blot (Figure 6A) and increased *hTERT* and *KRAS* expression by qPCR (Supplmental figure 2A, B). Lentivirus transduced hNSCs continued to express the stem cell markers CD133, SOX2, Nestin and OLIG2 (Figure 6B). These cells proliferated at an increased rate (7.9 and 9.6% BrdU positive cells examining two separate subclones) compared to control normal cortex-derived hNSCs (Figure 6C, 2.7±1.6% BrdU positive cells, *p*<0.05 Student's *t-*tests comparing separate clones to control hNSC).

Because a failure to differentiate properly is a hallmark of cancer cells, we compared the differentiation potential of LIN28A/DNp53/hTERT/KRAS hNSC to that of normal cortex-derived hNSC. When differentiated in 2% serum medium, LIN28A/DNp53/hTERT/KRAS hNSCs had increased expression of the glial marker GFAP and decreased expression of the neuronal marker MAP2, as shown by immunofluorescence and western blot (Figure 6 D,E). We also observed a baseline bias toward glial differentiation as evidenced by the first panels on the western blot, which show that even under non-differentiating conditions, that LIN28A/DNp53/hTERT/KRAS hNSC have significant glial differentiation. This is consistent with findings in DNp53/hTERT hNSC, which similarly showed bias toward glial differentiation (Supplemental Figure 2 C, D), compared to control GFP-transduced cortex-derived hNSCs.

**Figure 7:**
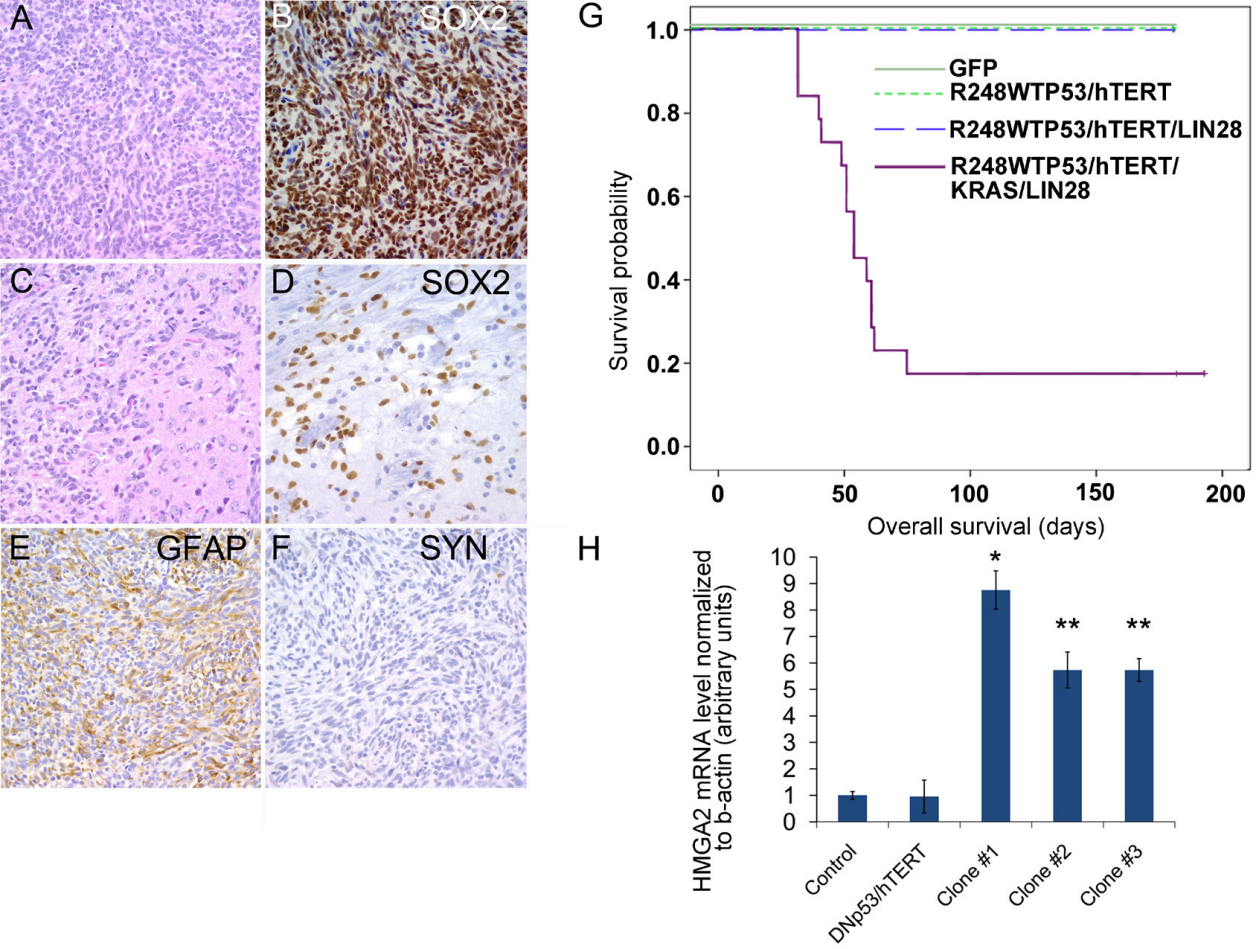
*LIN28A* promotes the transformation of human neural stem cells by dominant-negative *TP53*, constitutively active *KRAS*, and *hTERT* A. Hematoxylin and eosin stain of glial tumor formed by hNSC-DNp53/hTERT/KRAS/LIN28. B. Tumor xenografts formed by NSC-DNp53/hTERT/KRAS/LIN28A are positive for human specific SOX2 antibody by immunohistochemistry. C. Hematoxylin and eosin stain showing invasion of tumor (left) into surrounding brain parenchyma (right). D. Human specific SOX2 antibody identifies tumor cells invading deep into normal brain. E. Tumors are positive for GFAP by immunohistochemistry. F. Tumors are negative for Synaptophysin. 400X magnification for all images. G. Lethal glial tumors rapidly form in mice intracranially injected with hNSC-DNp53/hTERT/KRAS/LIN28A, while mice injected with control hNSC-GFP, hNSC-DNp53/hTERT or hNSC-DNp53/hTERT/LIN28A do not form tumors. Survival curve showing the decreased survival of mice injected with NSC-DNp53/hTERT/KRAS/LIN28A compared to controls. H. qPCR showing increased expression of *HMGA2* in NSC-DNp53/hTERT/KRAS/LIN28A, compared to control normal cortical neurospheres or NSC-DNp53/hTERT. Asterisk = ** p<0.01 versus control cortex and DNp53/hTERT hNSC, * p<0.05 versus control cortex and DNp53/hTERT hNSC by ANOVA.

To verify the role of LIN28A in the transformation of hNSCs, we tested the tumorigenicity *in vivo* of the LIN28A/DNp53/hTERT/KRAS expressing hNSCs and the hNSCs controls expressing DNp53/hTERT/KRAS, DNp53/hTERT/LIN28A, DNp53/hTERT, or GFP. Interestingly, DNp53/hTERT/KRAS cultures proliferated very poorly after infection, and we were unable to obtain sufficient cells for further study. Approximately eight weeks after orthotopic xenograft intracranial injection, 66.7% (12 of 18) of the mice injected with hNSC-LIN28A/DNp53/hTERT/ KRAS developed invasive brain tumors with glial phenotype (Figure 7A-F). In contrast, control injections including hNSC-DNp53/hTERT (10 mice), hNSC-LIN28A/DNp53/hTERT (5 mice) and hNSC-GFP (15 mice) did not generate tumors over 6 months (Figure 6G) (p=0.0003, Chi square hNSC-LIN28A/DNp53/hTERT/KRAS versus hNSC-GFP). Immunohistochemistry using human-specific SOX2 antibody identified tumor cells invading far into the normal mouse brain parenchyma (Figure 7A-D). Immunohistochemistry of hNSCs-LIN28A/DNp53/hTERT/KRAS xenografts showed LIN28A expression, activated pMAPK and accumulated TP53 (Supplemental Figure 3). These xenografts were positive for Nestin and GFAP and negative for Synaptophysin and exhibited increased proliferation as revealed by Ki67 staining (Figure 7 E, F and supplemental Figure 3). By qPCR, LIN28A/DNp53/hTERT/KRAS transduced neurospheres expressed more *HMGA2* mRNA than control GFP-transduced neurospheres (Figure 7H).

The lack of growth of hNSC after infection with DNp53/hTERT/KRAS implies LIN28A may play a facilitative role during the transformation of hNSCs. To further test this hypothesis, we used LIN28A-shRNA lentivirus to knock down LIN28A in hNSCs expressing LIN28A/DNP53/hTERT/KRAS. Strikingly, compared to empty vector or scramble shRNA, the cells infected with LIN28A-shRNA stopped proliferating, and we could not obtain enough cells for further study, consistent with the observation that hNSC- DNp53/hTERT/KRAS are not viable in long -term culture. The addition of *LIN28A* to DNp53/hTERT/KRAS appears to be a key element permitting *in vitro* proliferation and *in vitro* tumor formation in cells with high-level activation of the KRAS/MAP kinase pathway.

## DISCUSSION

The identification of pluripotency factors such as SOX2, OCT4, KLF4 and LIN28A in human cancer suggests a close relationship between tumor formation and the process of reprogramming mature cells to a more primitive type [1, 34]. Our findings that *LIN28A* is expressed in GBM and can facilitate and enhance tumor formation of GBM neurosphere cell lines further support the oncogenic potential of somatic cell reprogramming factors [1, 35]. The *LIN28A* homologue *LIN28B* promotes tumorigenicity in liver, ovarian, and colon progenitors and cancer cell lines [5, 36-38]. *LIN28A* is expressed in other CNS tumors, such as PNET, germ cell tumors and atypical teratoid rhabdoid tumors [6, 16, 17]. Our identification of *LIN28A* and *LIN28B* expression in a subset of GBM suggests a role for these reprogramming factors in GBM primary tumors.

In loss-of-function experiments, we demonstrate an on-going requirement of GBM cells for LIN28A to mediate invasion and growth. In gain-of-function experiments, expression of LIN28A in weakly tumorigenic GBM cell lines led to increased tumor formation and tumor size *in vivo* and increased GBM invasion *in vitro*. Increased tumorigenicity and invasion with increased LIN28A expression can be explained mechanistically by down-regulation of *let-7* microRNAs and up-regulation of *let-7* targets such as *HMGA2*, a chromatin modifying gene that is associated with numerous cancers [11, 12]. Consistent with these results, overexpression of *let-7g* can inhibit the proliferation of GBM neurosphere cell lines *in vitro* [39]. In our studies, expression of mature *let-7g* in *LIN28A* transduced GBM cells led to a decrease in *HMGA2* and reversed the *LIN28A*-mediated invasive phenotype *in vitro*.

HMGA2 is expressed in the developing brain and in neural stem cells, where it functions to suppress p16^INK4a^ [40]. The *pygmy* spontaneous genetic mouse mutant is deficient in *HMGA2* [41], and constitutive overexpression of LIN28A leads to mice of increased size (“mighty” mice) [42]. Human tall stature is associated with polymorphisms in *HMGA2* [43], suggesting a link between *LIN28A*, *let-7* microRNAs, and *HMGA2* and generalized growth. Consistent with this, we observe upregulation of HMGA2 in GBM cell lines and neural stem cells after LIN28A expression.

In diverse cancers, *HMGA2* promotes tumor invasiveness through activation of genes that suppress cell adhesion and promote migration [11, 12]. One well-known HMGA2 target is *SNAI1*, a zinc finger transcription factor which represses cell adhesion genes and promotes invasion [44]. Recently, the closely related transcription factor *SNAI2/SLUG* was found to be upregulated in a subset of GBM compared to normal brain and to promote invasion of GBM cells [45]. SNAI1 inhibition can decrease invasion and migration of glioblastoma cells *in vitro* [46]. SNAI1 can also cooperate with Hedgehog (SHH) and MYCN to bring about transformation of cerebellar neural precursor cells [47]. We observed increased *SNAI1* expression in GBM cell lines after forced expression of *LIN28A*. The corresponding decrease in expression of *let-7b* and *let-7g* after LIN28A expression suggests that increased LIN28A suppresses *let-7* microRNAs and de-represses HMGA2, which is then able to activate a program of increased pro-invasion genes, such as *SNAI1*. However, it is likely that *LIN28A*, as a master regulator of multiple microRNAs and downstream genes, can drive GBM tumorigenesis through a variety of mechanisms [15].

Most reports studying the transformation of neural stem cells have used mouse models or mouse-derived NSCs. Given the differences between the mouse and human central nervous system [48], we chose to use human NSCs to model the role of *LIN28A* in human GBM tumor formation. We introduced *LIN28A* together with a set of mutations commonly found in human primary GBM, including *R248WTP53*, *CA-KRAS*, *hTERT* into normal hNSC. This combination formed invasive tumors with evidence of glial differentiation, phenocopying GBM. While LIN28A/DNp53 /hTERT/KRAS hNSC rapidly formed aggressive orthotopic xenograft tumors, hNSC-DNP53/hTERT/KRAS did not survive in culture despite multiple attempts, and negative controls including hNSC-DNp53/hTERT, hNSC-LIN28A/DNp53/hTERT and hNSC-GFP did not generate tumors, suggesting that both *LIN28A* and activated *KRAS* play key roles in the transformation. The role of *LIN28A* in transformation is further strengthened by experiments showing that knockdown of *LIN28A* in hNSC-LIN28A/DNp53/hTERT/KRAS inhibited their growth, consistent with the results of *LIN28A* knockdown in glioma-derived neurosphere cell lines. LIN28A-related increased expression of HMGA2 may suppress p16^INK4a^ or other tumor suppressors that are induced by high-level activation of the RAS/BRAF/MAP kinase pathway in human neural stem cells. Our laboratory has recently shown that oncogene-induced senescence as evidenced by increased p16^INK4a^ expression is a key mechanism by which neural stem cells resist transformation by constitutive activation of the RAS/BRAF/MAP kinase pathway [49]. Studies of HMGA2-mediated repression of p16^INK4a^ and the oncogene-induced senescence program in human neural stem cells are on-going in our laboratory.

The experiments presented here indicate a role for *LIN28A* in the transformation of normal cells into aggressive glial tumors and an on-going requirement for LIN28A in GBM. By increasing the expression of multiple oncogenes simultaneously through repression of the *let-7* microRNAs, *LIN28A* can activate oncogenic programs that enhance glioblastoma aggressiveness. Our results show that *LIN28A* is expressed in GBM but not in normal human brain, and that *LIN28A* is important for the tumorigenicity of GBM cells and transformation of hNSCs, suggesting *LIN28A* might be a potential therapeutic target for GBM treatment.

## MATERIALS AND METHODS

### Cell culture

Human neural stem and progenitor cells were obtained from first trimester human fetal autopsy specimens in concordance with German law and Ethics Board evaluation. The study was also approved by the Johns Hopkins Institutional Review Board. Cells from cerebral cortex were microdissected and processed as described previously[18], then passaged in neurosphere media (30% Ham's F12 media, 70 % DMEM, 5% B27 regent (Invitrogen), 1% L-glutamine, 1% antibiotic-antimycotic, 5 micrograms/ml heparin, 20 ng/ml FGF, and 20 ng/ml EGF). Cells were split at high density after incubation with Accutase (Sigma-Aldrich) and gentle trituration. Human adult GBM cell lines HSR-GBM1 and 040622 cell lines (also known as HSR-GBM2), JHH-GBM14 have been previously described [19, 20]. The SF188 pediatric GBM cell line has been well characterized [21].

### DNA constructs and viral infection

The R248WTP53 plasmid (Addgene plasmid 16437) [22], and KRAS12V (Addgene plasmid 12544) [23] were subcloned separately into the lentiviral vector pWPI (Addgene plasmid 12254). Restriction digest and sequencing confirmed the fidelity of the subcloned sequences. Additional lentiviral constructs were LIN28A (Addgene Plasmid 16580) [24], hTERT (Addgene plasmid 12245) [25], pLKO.1 scramble shRNA (Addgene plasmid 1864) [26], and pLKO.1 empty vector (Addgene plasmid 10878) [27]. Lentivirus vectors containing short hairpins against LIN28A were purchased from Sigma (TRCN0000021800 and TRCN0000021803). Lentiviral particles were produced by transfecting 293T cells with VSVG envelope plasmid, delta 8.9 gag/pol plasmid and the plasmid of interest, as described previously, (20) using Fugene (Roche) per the manufacturer's instructions. After 24 hours, the media was changed to FGF/EGF neurosphere media, and supernatants were collected at 48 and 72 hours. These supernatants were pooled and passed through a 0.45 micron filter, then frozen at -80 C until needed. Human neural stem and progenitor cells growing as neurospheres were dissociated into single cells with Accutase and gentle trituration, and then incubated with lentiviral supernatants for 24 hours. For infection of human neural stem cells (hNSCs), combinations of lentivirus encoding LIN28A, R248WTP53, CA-KRAS, hTERT or GFP were added simultaneously. After approximately one week in culture, individual spheres were identified, and they were then individually placed into wells of a 24-well plate under selection with puromycin. Individual spheres were visually scored for GFP-positivity. Control cells were infected with pWPI empty vector, which constitutively expresses GFP. For infection of GBM cell lines, neurospheres were dissociated into single cells and lentivirus encoding LIN28A, LIN28A-shRNA empty vector or scramble shRNA was added. After approximately one week in culture, 2 ug/ml puromycin was used to select infected cells. For nucleofection, the Amaxa mouse neural stem cell kit was used (Lonzo).

### Immunofluorescence assays

For immunofluorescence assays, cells were either plated on Matrigel-coated Lab-Tek chamber slides (Nunc International) or were cytospun onto positively charged slides. After washing cells once with PBS, they were fixed with 4% paraformaldehyde for 15 minutes, permeabilized with 0.1% Triton/PBS, then blocked with 5% normal goat serum/PBST or 5% BSA/PBST, and incubated with the appropriate primary antibody. Primary antibodies included LIN28A (Cell Signaling Technology 3978, 1:100), GFAP (DAKO Z0334, 1:1000), MAP2 (Santa Cruz SC-20172 1:1000). After washing three times with PBST, cells were incubated for 45 minutes in the dark with the appropriate Cy-2 or Cy-3 conjugated secondary antibody (Jackson Immunoresearch). Cells were counterstained with DAPI and mounted with anti-fade (Vectastain).

### Assays of cell proliferation

BrdU assays were performed as described previously [28]. Anti-BrdU antibody was used as per the manufacturer's directions (Sigma B2531) at 1:100 dilution and visualized as described above.

For relative cell growth assay, cells were seeded in 96-well plates in triplicate at densities of 5000 cells per well. Cell proliferation was monitored at 0, 2, 4 and 7 days using the colorimetric CellTiter 96 MTS assay (Promega, Madison, WI).

### Colony formation in soft agarose

A 2X concentration of the neurosphere media was prepared and mixed 1:1 with 1 percent melted agarose (Invitrogen) in water to make bottom agar, which was used to coat each well of a 6-well plate. Cells were incubated in Accutase (Sigma) and triturated by pipetting through a P1000 pipette to single cell density and placed into the top agarose media/soft agarose mixture (0.5 %) and immediately plated into 6-well plates at a density of 5,000 cells/well in 1.5ml of agarose. After the agarose gelled, 2 ml of media was placed into each well. Media was changed every 7 days, and the cultures were incubated for 4 to 6 weeks. Colonies were visualized by staining with nitroblue tetrazolium (NBT) in a tissue culture incubator overnight at 37 C. Colonies greater than 50 microns in diameter were scanned and counted using MCID Elite software (Cambridge, England, UK).

### Boyden chamber transwell invasion assay

Boyden chambers (Falcon) were pre-coated with Matrigel (Invitrogen) at 1:100 dilution overnight. Cells were plated in the upper chamber in low growth factor media (1% serum) while the bottom chamber contained 10% serum. After 24 hour incubation, the cells remaining on top chamber were removed with a cotton-tipped applicator, and cells on the bottom of the membrane were fixed with 80% methanol and counterstained with hematoxylin. Cells were photographed at high power (200X) and three separate high power fields were counted.

### Quantitative RT-PCR

RNA levels were analyzed by real-time PCR analysis performed in triplicate with SYBR Green reagents (Applied Biosystems, Foster City, CA) according to the manufacturer's instructions on an I-Cycler IQ5 real-time detection system (Bio-Rad, Hercules, CA). The delta/delta cT method was used to determine expression levels, and all values were normalized to actin or 18S. Primer sequences were as follows: human LIN28A forward: CGGGCATCTGTAAGT, reverse: CAGACCCTTGGCTGA; human TERT forward: TGACACCTCACCTCACCCAC, reverse CACTGTCTTCCGCAAGTTCAC; human HMGA2 forward GCGCCTCAGAAGAGAGGAC, reverse: GGTCTCTTAGGAGAGGGCTCA; human SNAI1 forward: CATCCTTCTCACTGCCATGGA, reverse: AGGCAGAGGACACAGAACCAGA;human β-actin forward: CCCAGCACAATGAAGATCAA, reverse: GATCCACACGGAGTACTTG; human 18S forward: GTAACCCGTTGAACCCCATT, reverse: CCATCCAATCGGTAGTAGCG.

For detection of microRNAs, mature *let-7a*, *b* and *g* were quantified using a predesigned TaqMan MicroRNA Assay (Applied Biosystems) according to the manufacturer's instructions. An 18S rRNA primer was used as a normalizing control.

### TCGA Dataset analysis

Z-normalized mRNA or miRNA expression data from 503 glioblastomas with gene expression data and 426 tumors with miRNA expression data from the TCGA [29] were downloaded from the cBio Cancer Genomics Portal (http://www.cbioportal.org/). To evaluate the relationship between expression of different mRNAs or miRNAs, expression values were graphed as scatter plots and a best fit line derived using linear regression. The correlation of expression values was determined using Pearson's correlation coefficient. Significance was set at the p≤0.05. To determine pathway activation, a threshold Z-score of 1.5 was chosen and the TCGA dataset was interrogated for LIN28A, LIN28B, and HMGA2.

### Western blotting

Western blotting was performed as described [28], Antibodies were used as per the manufacturer's instructions and were as follows: LIN28A (1:1000; Cell Signal #3978); Nestin (1:2000; Millipore, #MAB5326), SOX-2 (1:500; Santa Cruz #SC17320), OLIG2 (1:2500; Millipore AB9610), CD133 (1:400; Abcam #AB19898), phosphor-MAPK (1:1000; Cell Signal #4376); MAPK (1:1000; Cell Signal #9102); TP53 (1:10,000; Sigma P 5813); MAP2 (1:5000; Santa Cruz #SC-20172); GFAP (1:5000; DAKO #Z0334); HMGA2 (1:1000; BioTek, #59170AP); glyceraldehyde-3-phosphate dehydrogenase (clone 6C5, 1:500,000; Research Diagnostics, Concord, MA).

### Intracranial xenograft tumors

For intracranial xenografts, injection guide holes were produced in anesthetized animals by twirling an 18-gauge beveled needle to provide access to the intracranial space. 1x10^5^ viable cells in 5 μl of growth medium were used for intracranial injection into the right striatum by stereotactic injection through a needle connected to a Hamilton syringe. The following coordinates were used: antero-posterior =-3mm; medio-lateral=2 mm; dorso-ventral =3 mm. The skin was sutured following injection. Animals were monitored daily for signs of distress suggestive of intracranial mass lesion (cachexia, neurologic deficits, poor grooming) and were sacrificed at that time. Xenograft tumors were paraffin-embedded and processed for immunohistochemistry by the Johns Hopkins Histopathology Core.

### Immunohistochemistry

Glioma tissue microarrays (TMAs) were constructed using tumor samples obtained from 1977 to 2009, and normal control tissue from autopsy specimens retrieved from the archives of the Johns Hopkins Hospital Department of Pathology following appropriate institutional review board approval. Criteria for inclusion included a diagnosis of invasive glioma and the presence of adequate tissue in the original paraffin block. Formalin-fixed, paraffin-embedded tissue was utilized to construct TMAs according to standard procedures at the Johns Hopkins tissue microarray core facility. For each tumor, four cores measuring 0.6 mm in diameter were used per array. Two neuropathologists independently determined tumor classification and cellularity.

Immunohistochemistry was performed on deparaffinized sections of brain xenografts and tissue microarrays containing cores from 135 gliomas. In brief, after antigen retrieval using Antigen Unmasking Solution (Vector Laboratories, Burlingame, CA), sections were incubated overnight at 4°C with primary antibodies. The following primary antibodies were used: LIN28A (1:100; Cell Signaling Technologies #3978); human specific NESTIN (1:500; Millipore, #MAB5326); GFAP (1:1000; DAKO #Z0334); Synaptophysin (1:50; DAKO #A0010); phospho-MAP kinase (1:100; Cell Signaling Technologies #4376); MAPK (1:100; Cell Signaling Technologies #9102); human specific SOX2 (1:50; Cell Signal #3579); MYC (1:200, Epitomics,# 1472-1) was diluted 1:200 in PBS TP53 (1:1000; Sigma P 5813); and Ki67 (1:100; Santa Cruz # SC-15402).

For LIN28A immunohistochemistry, cytoplasmic staining of LIN28A was scored as “strong” (2+) if in 10% or more of tumor cells it was as intense as in positive control spermatogonia, moderate (1+) if it was clearly positive but less intense than in these germ cells, and negative if it had only blush/trace or no immunoreactivity. SOX2 immunohistochemistry was scored as 2+ if 50% or more of tumors had nuclear positivity, 1+ if greater than 5% but less than 50% of cells had nuclear positivity, and negative if there was no immunoreactivity. MYC immunohistochemistry was graded as 0 if there was no reactivity, 1+ if weak nuclear staining in in less than 75% of tumor cells, 2+ if moderate or intense staining in less than 75% of cells, and 3+ if there was weak, moderate or strong staining in more than 75% of cells.

### Statistical Analysis

Statistical tests were performed using GraphPad Prism (GraphPad Software, San Diego California USA) or Excel (Microsoft). All tests were two sided unless otherwise indicated, and p values less than 0.05 were considered significant unless otherwise noted. In selected cases ANOVA was performed with Tukey multiple comparison adjustment, as indicated.

## SUPPLEMENTARY FIGURES


