# Supplementary material for: LIN28A facilitates the transformation of human neural stem cells and promotes glioblastoma tumorigenesis through a pro-invasive genetic program
Source: Oncotarget. 2013 Jul 6;4(7):1050–64. doi: 10.18632/oncotarget.1131 (PMC3759665; doi:10.18632/oncotarget.1131)
Supplement: Supplementary file 1 [file oncotarget-04-1050-s001.pdf]

LIN28A facilitates the transformation of human neural stem cells and promotes glioblastoma tumorigenesis through a pro-invasive genetic program-Mao et al

S1A. Western blot showing increased expression of LIN28A in LIN28A lentivirally transduced 040622 cells compared to GFP lentivirus-transduced cells.

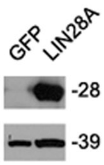

S1B. Hematoxylin and eosin stain of 040622-LIN28A tumor, showing invasion of brain parenchyma by tumor cells. Magnification = 200X.

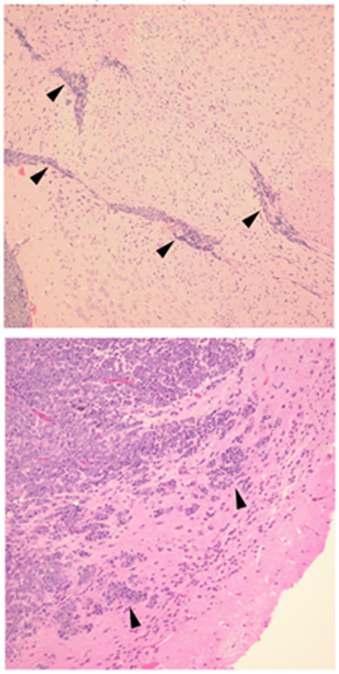

S1C. Immunohistochemistry of xenograft tumors formed by JHH-GBM14-LIN28A.

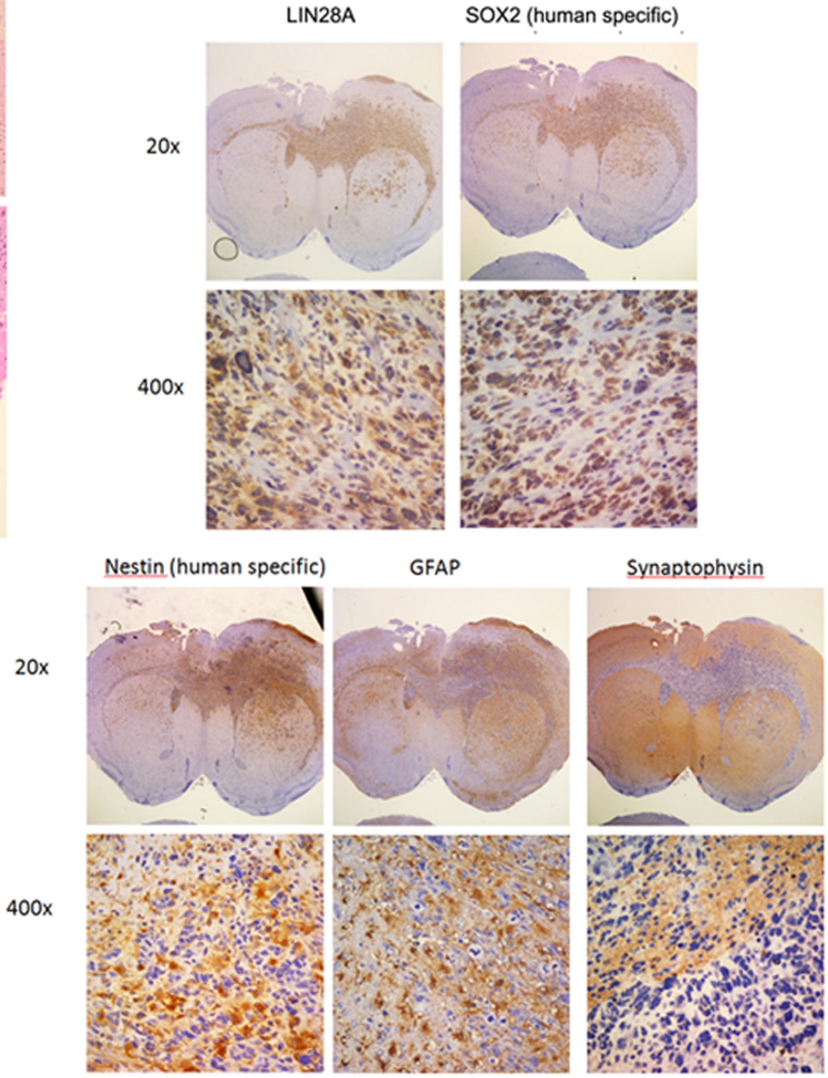

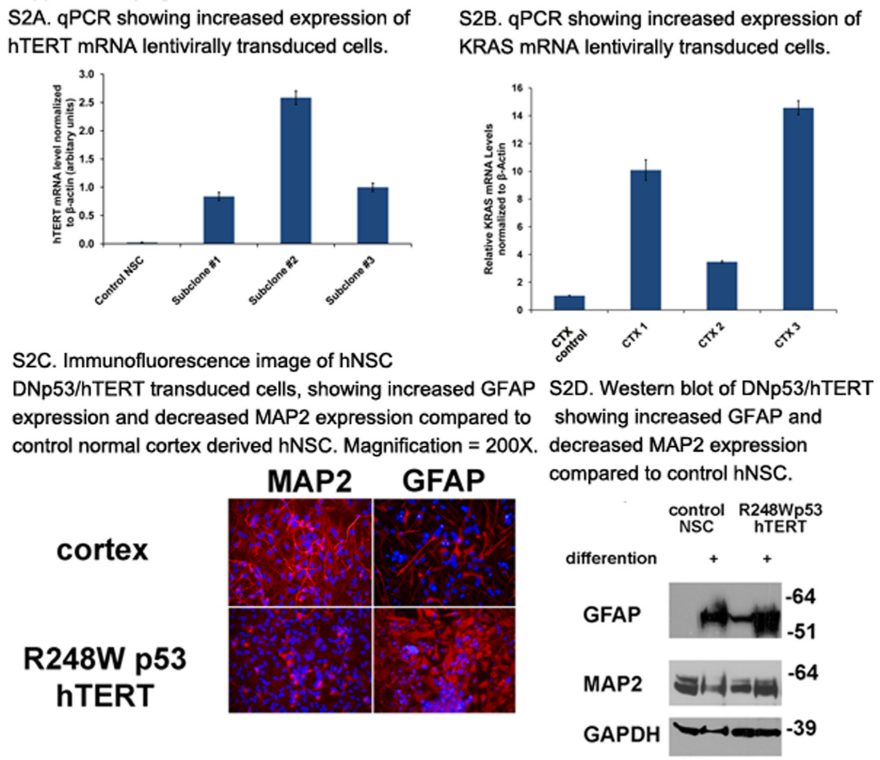

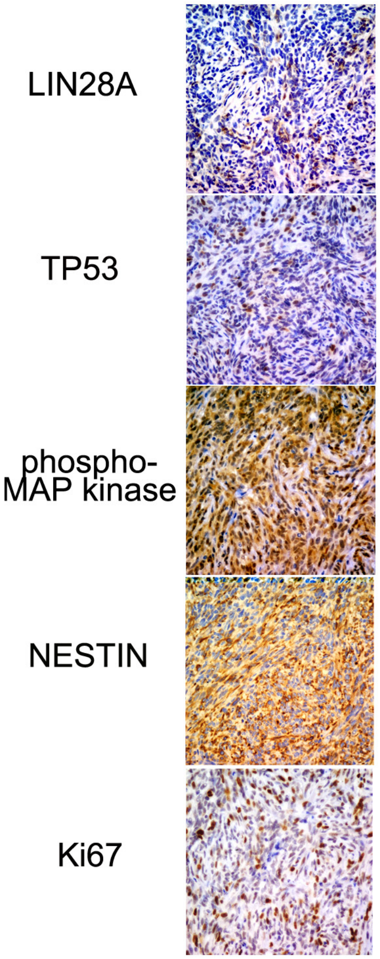

Supplemental figure 3: Immunohistochemistry showing that brain tumor xenografts formed by NSC-DNTP53/hTERT/KRAS/LIN28A express LIN28A, phospho-MAPK, TP53, Nestin, and Ki67. Magnification = 400X.
